# Longitudinal associations between BMI changes and musculoskeletal pain in older European males and females

**DOI:** 10.1038/s41366-026-02063-w

**Published:** 2026-04-08

**Authors:** Aisha Alayna Brown, Jesper Lykkegaard, Jonas Bloch Thorlund, Sören Möller, Jens Søndergaard, Linda Juel Ahrenfeldt

**Affiliations:** 1 Research Unit of General Practice, Department of Public Health, University of Southern Denmark, Odense-Esbjerg, Denmark; 2 Centre for Muscle and Joint Health, Department of Sports Science and Clinical Biomechanics, University of Southern Denmark, Odense, Denmark; 3 Research Unit for Epidemiology, Biostatistics and Biodemography, Department of Public Health, University of Southern Denmark, Odense, Denmark

**Keywords:** Risk factors, Obesity, Epidemiology, Lifestyle modification

## Abstract

**Background:**

Musculoskeletal pain affects nearly 200 million European citizens and is a leading cause of disability. This study examines the associations between body mass index (BMI) trajectories over time and self-reported musculoskeletal pain, and explores how these associations differ by sex.

**Methods:**

We conducted a longitudinal cohort study using data from waves 5–9 (2013–2022) of the Survey of Health, Ageing and Retirement in Europe. A total of 73,469 participants (mean age 70 years; 56% female) contributed at least two BMI measurements across study waves. Baseline and follow-up BMI values were categorized into sex-stratified quartiles, resulting in 16 distinct BMI trajectories. Associations between these trajectories and incident back, hip, knee, and other joint pain were examined using mixed-effects logistic regression models, adjusted for age, region, education, income, household partnership, comorbidities, and smoking. Stable low BMI served as the reference group.

**Results:**

Compared with stable low BMI trajectory, both upward and downward BMI trajectories, as well as stable high BMI, were associated with increased odds of musculoskeletal pain, with the strongest associations observed for stable high BMI. Stable high or changing BMI showed consistent associations with incident back, hip, and knee pain, while associations with other joint pain were weaker and less consistent. Overall, the associations were stronger for females than for males, but sex differences were most pronounced for hip and knee pain. Notably, maintaining a stable high BMI was associated with one of the highest odds of incident knee pain in females (odds ratio = 4.17, CI = 3.33–5.23).

**Conclusions:**

Elevated BMI at any time point was associated with increased odds of musculoskeletal pain, even among individuals who lost weight. The findings suggest that the cumulative duration of exposure to high BMI may be a critical factor in the development and prevention of musculoskeletal pain.

## Introduction

The association between body mass index (BMI) and musculoskeletal pain is well-documented, especially for weight-bearing joints. However, knowledge on the impact of weight changes over time on the development of musculoskeletal pain is still lacking, and the disease burden from musculoskeletal pain has increased substantially in the last decades [1]. About 200 million European citizens live with musculoskeletal pain [2]. Consequently, musculoskeletal pain contributes to a growing economic burden on society and significantly diminishes the quality of life and mental well-being of affected individuals [3]. Given the scale of this burden, understanding the underlying contributors, particularly the role of obesity in musculoskeletal pain, is essential for both prevention and treatment efforts. The double burden of obesity and musculoskeletal pain is evident, as 7.3 million years lost to disability (YLDs) were caused by high BMI in people with musculoskeletal pain [4].

Obesity, defined as a BMI of 30 kg/m² or higher [5], has been implicated directly in the symptomology of musculoskeletal pain, with research indicating that obesity can exacerbate the severity of musculoskeletal pain and potentially play a direct etiological role, leading to increased pain and disability [6]. In weight-bearing joints, the added pressure on the joints from having a BMI over 30 kg/m² is associated with a higher likelihood of developing musculoskeletal pain [7–9]. Furthermore, BMI over 30 kg/m² is also associated with pain in non-weight-bearing joints such as those in the arms and hands, suggesting that there may be a contributing factors beyond biomechanical stress [7]. While the mechanism behind this association is not yet elucidated, inflammation is a likely culprit. It is proposed that high body fat can contribute to low-grade inflammation, which in turn acts on various musculoskeletal pathways, causing damage to cartilage, joints, bones, and muscle tissue [6, 10]. This is a process that feeds back on itself, as obesity can lead to musculoskeletal issues, making maintaining an adequate level of exercise more difficult, and encouraging weight gain [6].

The literature has shown that BMI over 25 kg/m² is a modifiable risk factor for musculoskeletal pain [11–13]. Despite the growing burden of musculoskeletal pain, few studies have examined the long-term effects of BMI on musculoskeletal pain. While higher BMI is consistently associated with a greater risk of developing and sustaining musculoskeletal pain, particularly in working populations [11, 12], these findings are largely based on short-term or population-specific studies. Emerging longitudinal research highlights a bidirectional relationship between BMI and musculoskeletal pain over extended periods of time, suggesting that different BMI trajectories carry varying levels of risk depending on the pain site [14]. In their 2022 study, Radojčić et al. examined BMI trajectories over 19 years in postmenopausal women aged 45–64 and found that a BMI trajectory of 27–34 kg/m² was associated with knee and multi-site pain, while a BMI trajectory of 33–38 kg/m² conferred higher risk for hip and knee pain, compared to normal-weight controls [14]. These findings suggest that not all weight trajectories pose equal risk, raising the question of whether downward changes in BMI may confer greater protective effects compared to stable or upward trends, even when average BMI levels are similar. This points to BMI not only as a modifiable risk factor, but also to the potential importance of the BMI changes over time in shaping musculoskeletal outcomes. However, the extent to which these patterns hold true across different populations and timeframes remains unclear.

Sex differences further complicate this relationship. Females not only report higher rates of musculoskeletal pain [15–17] but also exhibit higher sensitivity in regards to pain perception [18, 19] and, on average, have a greater body fat percentage [20]. A cross-sectional analysis of 61,157 participants from the Survey of Health, Ageing, and Retirement (SHARE) across 14 European countries found that 35.7% of participants experienced chronic musculoskeletal pain, with a disproportionately higher prevalence among females (41.3%) than among males (29.1%) [21]. We aim to build on these and other longitudinal findings [11, 14, 21] by using SHARE data to investigate whether changes in BMI over time are associated with incident musculoskeletal pain in males and females. We hypothesized that high and upward BMI trajectories will be associated with greater odds of musculoskeletal pain, while downward BMI trajectories are expected to be associated with lower odds of musculoskeletal pain compared to the stable low group. These associations are anticipated to be more pronounced in females than in males.

## Methods and materials

### Setting and study population

This study is a longitudinal observational study utilizing data from SHARE [22], a panel survey that collects data on health, socio-economic status, and social and family networks of participants aged 50 years and older across Europe every two years. SHARE includes data from 28 European countries and Israel (excluding participants incarcerated, hospitalized, or living outside the country during the survey period), as well as their spouses or partners. Countries in SHARE used probability-based sampling methods to determine nationally representative samples. Eligible households were recruited using country-specific sampling frames.

Interviews are conducted through computer-assisted personal interviewing and carried out by trained interviewers. While interviews are primarily conducted with the same participants in each wave, new participants are also regularly included. Data collection includes both self-reported and objective health measures, including anthropometrics, and is conducted through computer-assisted personal interviews. Informed consent procedures are in place in each participating country [23]. In this study, we used data from SHARE waves 5–9 (2013–2022) including data from 27 European countries. Only participants taking part in at least two waves of SHARE with information on musculoskeletal pain at the first wave in which the participant took part (baseline) were included. We excluded participants with missing age (4 observations) and those lacking data on BMI at baseline and/or at least one follow-up BMI (2,278 observations). Moreover, based on prior research [24], we excluded participants with a height greater than 2.14 m (1,671 observations) or less than 1.21 m (232 observations), or a weight less than 20 kg (4,131 observations). It resulted in a population of 73,469 participants corresponding to 212,343 observations. The mean time between baseline and follow-up was 4.8 years.

### BMI and BMI changes

BMI was calculated using self-reported height and weight. We divided baseline and follow-up BMI values into sex-stratified quartiles, resulting in 16 possible trajectories based on participant’s quartile positions at both time points (i.e., the baseline wave and the follow-up wave(s)), which were grouped into downward trajectories (2-1, 3-1, 4-1, 3-2, 4-2, 4-3), upward trajectories (1-2, 2-3, 3-4, 1-3, 2-4, 1-4), and stable trajectories (1-1, 2-2, 3-3, 4-4), for ease of comparison.

The use of sex-stratified quartiles to define BMI trajectories allowed for a categorization of BMI that reflected the distribution within our specific sample and ensured that trajectory groups were balanced in size, thereby improving statistical power and comparability. Furthermore, quartile-based trajectories captured relative changes in BMI over time, enabling us to examine whether upward or downward movement within the population distribution, rather than absolute BMI thresholds, was related to musculoskeletal pain odds.

### Musculoskeletal pain

Information on musculoskeletal pain was obtained from follow-up wave(s). Participants were first asked the question by the interviewer: “Are you troubled with pain?”. Thereafter, pain was assessed with the question: “In which parts of the body do you feel pain?”. Response options included: “back”, “hips”, “knees”, “other joints”, “mouth/teeth”, “other parts of the body, but not joints”, and “all over”. Due to the focus on musculoskeletal pain in this study, we only included questions pertaining to musculoskeletal pain (i.e., back, hips, knees, and other joints).

### Sociodemographic variables

Sociodemographic variables were obtained from follow-up wave(s). Confounders included sex, age, European region, partner in household, highest obtained education, household income, comorbidity index (diagnosis of heart attack, hypertension, high cholesterol, stroke, diabetes, lung disease, cancer, ulcer, and Parkinson’s Disease on a scale of 0–3+), and smoking history.

Based on classifications used in prior SHARE studies [25–27], the 27 European countries were combined into four regions: Northern Europe (Denmark, Sweden, and Finland), Western Europe (Austria, Germany, the Netherlands, France, Switzerland, Belgium, and Luxembourg), Southern Europe (Spain, Italy, Greece, and Portugal, Cyprus, Malta, Lithuania, Bulgaria), and Eastern Europe (Czech Republic, Poland, Hungary, Slovenia, Estonia, Croatia, Latvia, Romania, and Slovakia).

### Statistical methods

To assess the associations between BMI trajectories and incident musculoskeletal pain, we applied mixed-effects logistic regression models to estimate odds ratios (ORs) with 95% confidence intervals (CIs), using the 1–1 trajectory (stable low BMI) as the reference group. The models accounted for repeated observations, as participants could contribute data from multiple follow-up waves by including a random intercept for each participant. All analyses were adjusted for age, European region, education, household income, partner in household, comorbidity index, and smoking, based on Directed Acyclic Graph Theory (Supplementary Fig. 1) [28, 29]. To account for documented sex differences in musculoskeletal conditions [18, 21], analyses were stratified by sex. Furthermore, participants reporting back, hip, knee, or other joint pain at baseline were excluded from the corresponding outcome-specific analysis (e.g., those with back pain at baseline were excluded from the analysis of backpain). As a sensitivity analysis, we repeated the main analysis using only the first follow-up wave for each participant to assess whether results were influenced by the inclusion of multiple follow-up observations. All model assumptions were met.

The dataset was created in Stata version 18.0, while all analyses were carried out in R version 4.4.2.

## Results

This study included 73,469 participants, of which 41,141 (56.0%) were females and 32,328 (44.0%) were males, with a mean age of 70 years (standard deviation (SD) = 9.2). There were more females than males who experienced musculoskeletal pain (51.3% vs. 39.5%). Males were more likely to live with a partner (80.2% vs. 60.1%), be highly educated (26.0% vs. 22.1%), have three or more comorbidities (10.7% vs. 9.2%), and be a current or former smoker (51.9% vs. 30.5%) than females. The highest percentage of participants with musculoskeletal pain were found in Eastern Europe (51.9%) and the lowest was in Northern Europe (36.7%) (Table 1).

**Table 1:** Demographic characteristics of study participants.

	Overall	Males	Females	Northern	Western	Southern	Eastern
*N* (%)	73,469 (100.0)	32,328 (44.0)	41,141 (56.0)	8455 (11.5)	24,619 (33.5)	15,254 (20.8)	25,141 (34.2)
Age (mean (SD))	70.2 (9.2)	70.3 (8.9)	70.09 (9.43)	70.4 (9.2)	69.9 (9.3)	70.9 (9.3)	70.0 (9.0)
Starting BMI (mean (SD))	27.1 (4.6)	27.3 (4.0)	26.83 (4.95)	26.1 (4.1)	26.6 (4.6)	27.1 (4.3)	28.0 (4.7)
Any Pain, Yes (*n* (%))	64,158 (46.2)	23,876 (39.5)	40,282 (51.3)	6759 (36.7)	21,335 (42.1)	14,499 (51.4)	21,565 (51.9)
Back Pain, Yes (*n* (%))	32,965 (23.7)	12,041 (19.9)	20,924 (26.7)	3020 (16.4)	11,195 (22.1)	7425 (26.3)	11,325 (27.2)
Hip Pain, Yes (*n* (%))	14,876 (10.7)	4815 (8.0)	10,061 (12.8)	1655 (9.0)	4528 (8.9)	2980 (10.6)	5713 (13.7)
Knee Pain, Yes (*n* (%))	25,742 (18.5)	8889 (14.7)	16,853 (21.5)	2312 (12.6)	7672 (15.1)	6664 (23.6)	9094 (21.9)
Other Joint Pain, Yes (*n* (%))	21,699 (15.6)	7206 (11.9)	14,493 (18.5)	2078 (11.3)	7327 (14.5)	5319 (18.9)	6975 (16.8)
Lives with Partner (*n* (%))	95,593 (68.8)	48,454 (80.2)	47,139 (60.1)	13,092 (71.1)	34,545 (68.1)	20,841 (73.9)	27,115 (65.2)
Education Level (*n* (%))							
Low	48,869 (35.2)	18,955 (31.4)	29,914 (38.1)	4435 (24.1)	13,589 (26.8)	18,742 (66.5)	12,103 (29.1)
Middle	56,160 (40.4)	25,412 (42.1)	30,748 (39.2)	6575 (35.7)	21,911 (43.2)	5856 (20.8)	21,818 (52.5)
High	33,039 (23.8)	15,697 (26.0)	17,342 (22.1)	7280 (39.6)	14,782 (29.2)	3454 (12.3)	7523 (18.1)
Income (median (IQR))	19,700 (10,000.0–38,316.5)	22,700 (11,734.7–42,802.2)	17,812 (8880.0–34,852.0)	38,720 (24,872.6–58,338.7)	35,620 (22,500.0–55,903.0)	14,398 (8760.0–22,077.6)	9048 (5,581.0–13,803.6)
Comorbidity Index (*n* (%))							
0	47,675 (34.3)	19,652 (32.5)	28,023 (35.7)	7738 (42.0)	19,286 (38.0)	8508 (30.2)	12,143 (29.2)
1	43,524 (31.3)	18,876 (31.2)	24,648 (31.4)	5541 (30.1)	15,958 (31.5)	8968 (31.8)	13,057 (31.4)
2	28,266 (20.4)	12,551 (20.8)	15,715 (20.0)	3086 (16.8)	9459 (18.7)	6347 (22.5)	9374 (22.6)
3+	13,695 (9.9)	6,464 (10.7)	7,231 (9.2)	1497 (8.1)	4164 (8.2)	3179 (11.3)	4855 (11.7)
Ever Smoked, Yes (*n* (%))	55,275 (39.8)	31,340 (51.9)	23,935 (30.5)	9665 (52.5)	21,450 (42.3)	8741 (31.0)	15,419 (37.1)

The count of participants (*N*) is based on individuals; the rest of the table is number of observations.

Mean BMI and number of participants for each baseline quartile stratified by sex is shown in Table 2. In males, the mean BMI for quartiles 1–4 were 22.89 (SD = 1.51), 25.71 (SD = 0.64), 28.10 (SD = 0.77), and 32.77 (SD = 3.31), respectively. In females, the mean BMIs for quartiles 1–4 were 21.39 (SD = 1.61), 24.80 (SD = 0.79), 27.80 (SD = 0.99), and 33.64 (SD = 3.83), respectively. Males had slightly higher mean BMI for all quartiles except quartile four.

**Table 2:** Summary of BMI for quartiles at baseline.

Quartiles	Observations	Mean BMI	SD	Range
Males				
1 (lowest)	8082	22.89	1.51	13.8–24.6
2	8082	25.71	0.64	24.6–26.8
3	8082	28.10	0.77	26.8–29.4
4 (highest)	8082	32.77	3.31	29.4–80.0
Females				
1 (lowest)	10,286	21.39	1.61	12.5–23.4
2	10,285	24.80	0.79	23.4–26.2
3	10,285	27.80	0.99	26.2–29.7
4 (highest)	10,285	33.64	3.83	29.7–73.5

### Main analysis

**Fig. 1:**
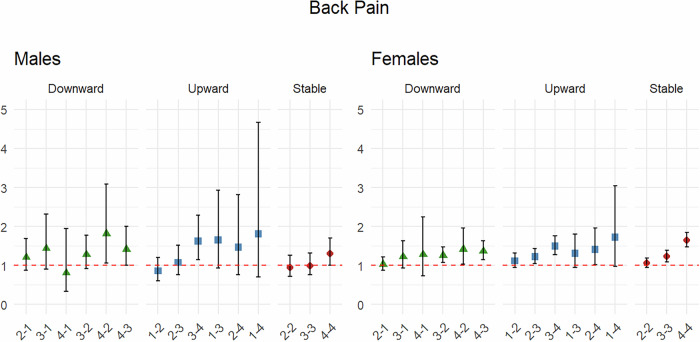
Odds of incident back pain for males and females for the 16 possible BMI trajectories.

Overall, in both males and females, higher BMI levels over time were associated with an increased odds of incident back pain compared to the stable low BMI reference group. Regarding weight changes, individuals who reduced their BMI from high to moderate or low levels exhibited elevated odds of incident back pain compared to those who had maintained a stable low BMI. This association was most pronounced in males, where being in the highest BMI quartile at either baseline or follow-up was associated with increased odds of incident back pain, regardless of weight loss at follow-up. A similar pattern was observed in females, though the associations were generally weaker. Furthermore, among females, having a stable high BMI between baseline and follow-up was associated with higher odds of incident back pain than having a stable low BMI (Fig. 1, Supplementary Table 1).

**Fig. 2:**
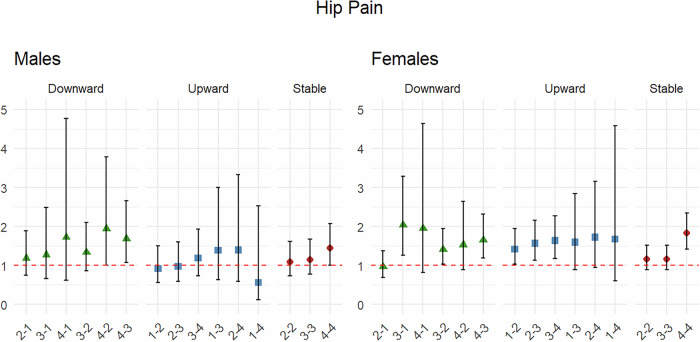
Odds of incident hip pain for males and females for the 16 possible BMI trajectories.

Among males, incident hip pain was associated with high BMI, regardless of whether the trajectory was upward or downward (4-4, 4-3, and 4-2). In females, associations were consistent across BMI trajectories, with particularly elevated odds of incident hip pain observed in those with downward trajectories and those with a stable high BMI (Fig. 2, Supplementary Table 1).

**Fig. 3:**
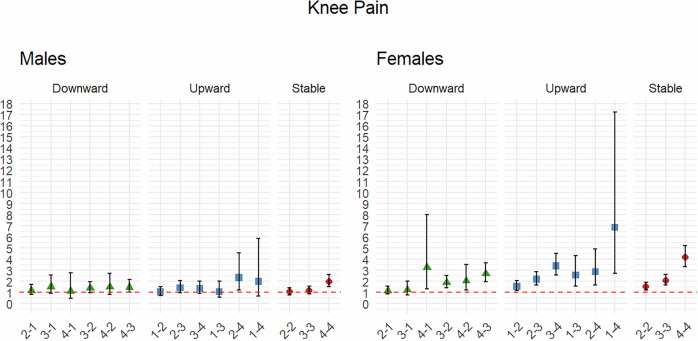
Odds of incident knee pain for males and females for the 16 possible BMI trajectories.

In males, knee pain was associated with two upward trajectories (2-4 and 2-3) and one downward trajectory (4-3), as well as with stable high BMI. In females, a broader range of BMI trajectories were associated with knee pain. This included upward shifts from low or moderate BMI to higher levels, as well as stable moderate and high BMI trajectories. One of the strongest associations was observed in females who had a stable high BMI trajectory (4–4), with over a fourfold increase in the odds of knee pain compared to those with stable low BMI (odds ratio = 4.17, CI = 3.33–5.23). Every BMI trajectory except for 2-1 and 3-1 was associated with increased odds of knee pain in females (Fig. 3, Supplementary Table 1).

**Fig. 4:**
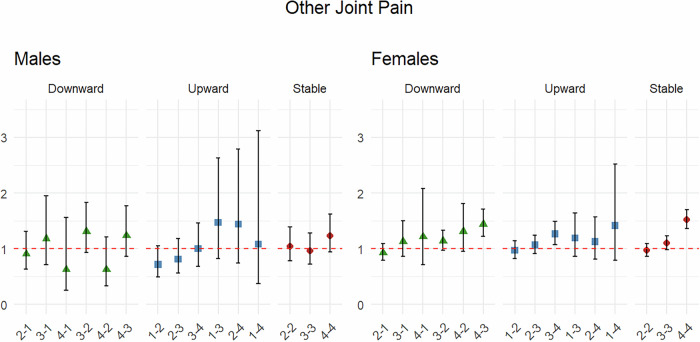
Odds of incident other joint pain for males and females for the 16 possible BMI trajectories.

In males, no BMI trajectories were associated with other joint pain. In females, three trajectories, one downward, one upward, and one stable high trajectory (4-3, 3-4, and 4-4, respectively) showed associations with other joint pain (Fig. 4, Supplementary Table 1).

A sensitivity analysis was performed, and results were consistent with the main findings across sexes and anatomical sites. These findings are presented in Supplementary Table 2 and Supplementary Figs. 2–5.

## Discussion

### Summary of key findings

In this large European cohort of adults aged 50 and older, changes in BMI over time were associated with incident musculoskeletal pain across several anatomical sites. Compared to individuals with stable low BMI (1-1), elevated odds of pain were observed not only for participants with stable high BMI, but also among those with increasing or decreasing BMI trajectories. These associations were the most consistent and strongest for knee pain, followed by back and hip pain. Associations with other joint pain were weaker and less consistent. Sex differences were evident. In females, high or changing BMI was consistently associated with increased odds of hip and knee pain, with the strongest association observed for stable high and increasing (1-4) BMI trajectories and incident knee pain. Back pain was associated with BMI changes in both sexes, but more strongly in males. Other joint pain showed no associations in males and less consistent associations in females. Overall, the associations were more pronounced and widespread in females.

### Comparison to other studies

We identified that of the investigated anatomical sites, incident knee pain had the strongest associations with BMI trajectories, specifically for participants with the stable high BMI and females. A previous longitudinal study by Radojčić et al. [14] investigating the association between BMI and musculoskeletal pain in postmenopausal females found that high BMI was associated with higher odds of knee pain than pain in other areas of the body. Specifically, they observed that participants with a BMI trajectory of 27–34 kg/m² had a bidirectional relationship with knee and multisite musculoskeletal pain, suggesting that knee pain can predict weight gain, and weight gain can predict knee pain. Further, they found that participants maintaining a BMI of 33–38 kg/m² had a unidirectional association with lower limb pain. In line with this, we observed that increasing BMI was more strongly associated with incident knee pain than pain in other anatomical sites. Even many decreasing BMI trajectories were associated with higher odds of incident knee pain compared to maintaining a stable low BMI, an association that was particularly pronounced in females. Overall, these findings suggest that having a high BMI at any time is associated with increased odds for incident musculoskeletal pain, regardless of subsequent weight loss, compared to maintaining a stable low BMI. This indicates that the duration of time spent with a high BMI may be an important factor for musculoskeletal pain prevention. However, these associations may be influenced by potential confounding factors such as physical activity levels or pre-existing joint conditions, which could not be fully accounted for in this analysis.

Several mechanisms have been proposed to explain how BMI affects musculoskeletal pain. One hypothesis is that high body fat drives low-grade inflammation, contributing to pain in non–weight-bearing regions [30], while excess weight directly increases mechanical stress on weight-bearing joints such as the back, hips, and knees [31]. Our data show the strongest and most consistent associations between BMI trajectories and pain in the back, knees, and hips, whereas associations with other joint pain, where the effect of mechanical load is not clear, were weaker and less consistent. Although research suggests that body fat percentage may mediate the link between BMI and musculoskeletal pain [32], this pathway remains unclear. Some studies report that weight loss reduces systemic inflammation and thus pain [33, 34]. However, Perera et al. [31] found no mediating effect of inflammatory markers on the association between BMI and back pain; instead, they observed a direct effect of BMI, total fat mass, and lean mass. Because our analysis did not include inflammatory markers, we could not address that pathway directly. However, our findings emphasize that both increasing BMI, stable high BMI, and decreasing BMI trajectories in many cases, were associated with pain in weight-bearing joints. Given the above considerations, one may expect a lower or no odds of incident musculoskeletal pain in downward trajectories, but this was not the case in our study. This discrepancy may be partly explained by our study design. Participants entered the study without musculoskeletal pain, whereas many previous studies enrolled participants who already had pain at baseline [31, 33, 34]. It is also possible that some participants lost weight because of treatment recommendations to help manage high pain levels they were already experiencing.

It has been well documented that females report more musculoskeletal pain than males [15–17]. This tendency is further exemplified in SHARE data, in which it was found that females with chronic musculoskeletal pain had a prevalence of 41.3% while males only had a prevalence of 29.1% [21]. We found not only that females were more likely to have pain in any of the body areas that were investigated, but also the association between BMI and musculoskeletal pain was generally stronger in females than in males. This could be attributed to naturally higher fat percentage that is found in females [20], hormonal differences, or differences in pain perception [18].

Our large sample size increases the likelihood of detecting small effect sizes that may not be clinically relevant. While some trajectories, such as stable high BMI, showed strong associations with musculoskeletal pain, particularly knee pain in females (OR = 4.17), other associations were more modest. These smaller effect sizes may reflect limited clinical impact. Interpreting these findings in the context of clinical practice requires caution, and future research should aim to identify thresholds of BMI change that are clinically meaningful for musculoskeletal health.

### Strengths and limitations

Major strengths of this study included the large sample size and longitudinal design, which allowed us to track BMI trajectories over time and examine their associations with musculoskeletal pain. The inclusion of multiple European regions and stratification by sex enhances the generalizability of our findings. However, several limitations should be noted. First, the observational nature of the study limits causal inference. Second, potential confounding by unmeasured variables such as dietary habits and body fat percentage, could not be addressed, introducing the possibility of residual confounding. Another unmeasured variable is the chronicity of musculoskeletal pain. Because this was not consistently collected between waves, we were unable to distinguish between acute and chronic musculoskeletal pain. Furthermore, our study could not differentiate between specific non-weight-bearing joints, limiting our ability to explore anatomical nuances.

Due to incomplete data across waves for some participants, we were unable to separate the timing of BMI and musculoskeletal pain measurements, as both were collected within the same wave. This limits our ability to determine the directionality of associations and raises the possibility of reverse causation. This issue is particularly relevant for participants in trajectories 4–1 and 4–2, who experienced substantial decreases in BMI from baseline to follow-up. Such weight loss may reflect underlying health conditions that could influence both BMI and pain outcomes. Although we adjusted for comorbidities to mitigate this concern, the absence of information on the timing of diagnoses prevented us from excluding participants with recent illnesses that may have contributed to weight loss.

The reliance on self-reported data also poses a chance for reporting bias [35], and sex differences in pain perception and reporting may complicate direct comparisons between males and females [18, 19, 21]. Finally, while the duration of exposure to high BMI may be a critical predictor of musculoskeletal pain [36], we lacked lifetime BMI data and were therefore limited to examining BMI changes over a restricted timeframe.

## Conclusion

This study highlights that both stable high BMI and changes in BMI over time are associated with incident musculoskeletal pain. Notably, our findings suggest that high BMI, regardless of weight loss, was associated with increased odds of incident musculoskeletal pain. These associations were most pronounced in females, particularly with knee pain. This indicates that prior exposure to excess weight may play a role in the development of musculoskeletal pain, and weight may not necessarily reduce these odds. These results underscore the complex relationship between weight dynamics and musculoskeletal health, suggesting that prevention efforts should focus not only on current BMI but also on limiting the duration of time spent with excess weight. Further research is needed to better understand the biological mechanisms underlying these associations, including the potential impacts of weight loss itself on musculoskeletal pain.

## Supplementary information

Supplementary Material

## Data Availability

Data from SHARE data is free of charge for scientific use globally http://www.share-project.org/data-access.html.

## Code availability

The code generated in this study is available from the corresponding author upon reasonable request.
