# Supplementary material for: Longitudinal associations between BMI changes and musculoskeletal pain in older European males and females
Source: Int J Obes (Lond). 2026 Apr 8;50(7):1486–93. doi: 10.1038/s41366-026-02063-w (PMC13391371; doi:10.1038/s41366-026-02063-w)

Supplementary Tables

Supplementary Table 1 - Longitudinal associations between BMI and the odds of musculoskeletal pain among middle-aged and older Europeans participating in at least two waves of SHARE 2013 and 2022, stratified by sex.

	males		females	
**Back Pain**				
**BMI Trajectories**	**Odds Ratio**	**Confidence Interval**	**Odds Ratio**	**Confidence Interval**
2-1	1.22	(0.87-1.69)	1.04	(0.88-1.22)
3-1	1.41	(0.88-2.25)	1.24	(0.93-1.64)
4-1	0.80	(0.33-1.93)	1.35	(0.77-2.36)
1-2	0.86	(0.61-1.21)	1.12	(0.95-1.31)
2-2	0.95	(0.72-1.25)	1.06	(0.94-1.19)
3-2	1.28	(0.92-1.77)	1.25	(1.07-1.47)
4-2	1.82	(1.07-3.10)	1.42	(1.03-1.96)
1-3	1.65	(0.93-2.93)	1.35	(0.98-1.85)
2-3	1.07	(0.76-1.51)	1.23	(1.06-1.43)
3-3	0.99	(0.76-1.31)	1.25	(1.11-1.40)
4-3	1.42	(1.01-2.00)	1.36	(1.14-1.62)
1-4	1.87	(0.73-4.80)	1.73	(0.98-3.05)
2-4	1.47	(0.77-2.80)	1.41	(1.02-1.96)
3-4	1.63	(1.16-2.29)	1.49	(1.27-1.75)
4-4	1.31	(1.01-1.70)	1.66	(1.48-1.86)
**Hip Pain**				
2-1	1.19	(0.75-1.89)	0.97	(0.68-1.37)
3-1	1.23	(0.63-2.40)	2.00	(1.24-3.22)
4-1	1.74	(0.63-4.80)	1.99	(0.84-4.75)
1-2	0.92	(0.56-1.50)	1.41	(1.03-1.94)
2-2	1.09	(0.74-1.61)	1.16	(0.89-1.51)
3-2	1.36	(0.87-2.12)	1.44	(1.05-1.97)
4-2	1.97	(1.01-3.83)	1.54	(0.89-2.65)
1-3	1.39	(0.64-3.00)	1.59	(0.89-2.85)
2-3	0.97	(0.59-1.60)	1.58	(1.15-2.17)
3-3	1.15	(0.78-1.68)	1.18	(0.91-1.53)
4-3	1.67	(1.06-2.63)	1.67	(1.20-2.34)
1-4	0.56	(0.12-2.52)	1.67	(0.61-4.59)
2-4	1.4	(0.59-3.32)	1.74	(0.95-3.18)
3-4	1.19	(0.73-1.93)	1.61	(1.15-2.24)
4-4	1.45	(1.01-2.08)	1.82	(1.42-2.34)
**Knee Pain**				
2-1	1.18	(0.82-1.70)	1.16	(0.86-1.56)
3-1	1.48	(0.88-2.50)	1.28	(0.80-2.06)
4-1	1.16	(0.47-2.85)	3.43	(1.39-8.51)
1-2	1.05	(0.73-1.53)	1.56	(1.17-2.07)
2-2	1.07	(0.79-1.45)	1.54	(1.22-1.94)
3-2	1.39	(0.98-1.98)	1.87	(1.41-2.49)
4-2	1.52	(0.84-2.73)	2.07	(1.21-3.54)

1-3	1.07	(0.57-2.02)	2.59	(1.54-4.33)
2-3	1.44	(1.00-2.07)	2.19	(1.67-2.88)
3-3	1.17	(0.87-1.58)	2.11	(1.68-2.64)
4-3	1.48	(1.02-2.14)	2.78	(2.05-3.77)
1-4	1.99	(0.68-5.84)	6.86	(2.72-17.30)
2-4	2.35	(1.21-4.56)	2.87	(1.68-4.90)
3-4	1.42	(0.97-2.07)	3.45	(2.58-4.60)
4-4	2.00	(1.51-2.65)	4.17	(3.33-5.23)
**Other Joint Pain**				
2-1	0.91	(0.64-1.31)	0.93	(0.79-1.09)
3-1	1.15	(0.7-1.91)	1.13	(0.85-1.48)
4-1	0.62	(0.25-1.54)	1.27	(0.74-2.17)
1-2	0.72	(0.49-1.05)	0.97	(0.82-1.15)
2-2	1.05	(0.79-1.40)	0.97	(0.87-1.09)
3-2	1.32	(0.94-1.84)	1.15	(0.99-1.35)
4-2	0.63	(0.33-1.21)	1.32	(0.96-1.81)
1-3	1.47	(0.82-2.63)	1.2	(0.87-1.66)
2-3	0.82	(0.56-1.19)	1.09	(0.93-1.27)
3-3	0.97	(0.73-1.29)	1.11	(0.98-1.24)
4-3	1.24	(0.86-1.77)	1.45	(1.23-1.72)
1-4	1.08	(0.38-3.11)	1.42	(0.80-2.53)
2-4	1.44	(0.75-2.79)	1.12	(0.80-1.55)
3-4	0.99	(0.68-1.44)	1.29	(1.09-1.51)
4-4	1.23	(0.94-1.62)	1.52	(1.36-1.70)

Adjusted for age, wave, region, Partner in household, education, household income, and history of diseases.
OR = Odds ratio, CI = confidence interval.

Supplementary Table 2 - Longitudinal associations between BMI and the odds of musculoskeletal pain among middle-aged and older Europeans based on their two most recent waves of participation in SHARE, stratified by sex.

	males		females	
**Back Pain**				
**BMI Trajectories**	**Odds Ratio**	**Confidence Interval**	**Odds Ratio**	**Confidence Interval**
2-1	0.95	(0.78-1.15)	1.02	(0.86-1.21)
3-1	1.08	(0.77-1.48)	1.04	(0.75-1.43)
4-1	1.09	(0.62-1.80)	1.09	(0.57-1.96)
1-2	1.04	(0.85-1.27)	1.03	(0.87-1.22)
2-2	0.94	(0.82-1.08)	1.07	(0.96-1.19)
3-2	1.07	(0.89-1.29)	1.16	(0.98-1.36)
4-2	1.26	(0.87-1.76)	1.38	(0.97-1.92)
1-3	0.98	(0.68-1.40)	1.17	(0.82-1.64)
2-3	1.14	(0.95-1.37)	1.12	(0.96-1.31)
3-3	1.14	(1.00-1.30)	1.17	(1.05-1.31)
4-3	1.26	(1.03-1.53)	1.22	(1.02-1.45)
1-4	1.47	(0.83-2.48)	1.64	(0.93-2.79)
2-4	1.24	(0.84-1.78)	1.29	(0.92-1.78)
3-4	1.37	(1.14-1.64)	1.39	(1.18-1.62)

4-4	1.21	(1.07-1.37)	1.38	(1.25-1.53)
**Hip Pain**	0.95	(0.78-1.15)		
2-1	1.08	(0.77-1.48)	1.21	(0.99-1.48)
3-1	1.09	(0.62-1.80)	1.27	(0.87-1.81)
4-1	1.04	(0.85-1.27)	1.81	(0.92-3.28)
1-2	0.94	(0.82-1.08)	1.16	(0.93-1.44)
2-2	1.07	(0.89-1.29)	1.20	(1.04-1.38)
3-2	1.26	(0.87-1.76)	1.16	(0.95-1.41)
4-2	0.98	(0.68-1.40)	1.18	(0.75-1.78)
1-3	1.14	(0.95-1.37)	1.76	(1.18-2.54)
2-3	1.14	(1.00-1.30)	1.43	(1.18-1.72)
3-3	1.26	(1.03-1.53)	1.28	(1.12-1.47)
4-3	1.47	(0.83-2.48)	1.38	(1.12-1.69)
1-4	1.24	(0.84-1.78)	2.31	(1.20-4.12)
2-4	1.37	(1.14-1.64)	1.60	(1.09-2.29)
3-4	1.21	(1.07-1.37)	1.52	(1.25-1.84)
4-4	0.95	(0.78-1.15)	1.70	(1.50-1.93)
**Knee Pain**				
2-1	1.12	(0.90-1.39)	1.33	(1.10-1.60)
3-1	1.05	(0.70-1.52)	1.48	(1.04-2.06)
4-1	1.57	(0.88-2.64)	1.67	(0.79-3.20)
1-2	1.40	(1.13-1.73)	1.40	(1.15-1.69)
2-2	1.08	(0.93-1.26)	1.46	(1.28-1.65)
3-2	1.20	(0.97-1.48)	1.71	(1.44-2.03)
4-2	1.62	(1.10-2.32)	2.48	(1.74-3.48)
1-3	1.07	(0.70-1.58)	2.15	(1.51-2.98)
2-3	1.63	(1.34-1.97)	1.97	(1.67-2.32)
3-3	1.29	(1.11-1.49)	1.80	(1.59-2.04)
4-3	1.41	(1.13-1.75)	2.00	(1.66-2.40)
1-4	1.71	(0.89-3.04)	3.81	(2.21-6.35)
2-4	1.99	(1.35-2.86)	3.07	(2.26-4.15)
3-4	1.44	(1.17-1.77)	2.38	(2.01-2.82)
4-4	1.76	(1.54-2.01)	2.66	(2.37-2.99)
**Other Joint Pain**				
2-1	0.77	(0.60-0.97)	0.95	(0.79-1.13)
3-1	0.92	(0.62-1.34)	1.08	(0.77-1.47)
4-1	1.10	(0.58-1.92)	1.02	(0.51-1.86)
1-2	0.86	(0.67-1.09)	1.03	(0.86-1.23)
2-2	1.04	(0.90-1.21)	1.02	(0.91-1.14)
3-2	1.13	(0.92-1.38)	1.15	(0.97-1.35)
4-2	1.02	(0.66-1.52)	1.82	(1.30-2.51)
1-3	0.90	(0.58-1.34)	1.44	(1.02-2.00)
2-3	0.93	(0.75-1.15)	1.02	(0.87-1.20)
3-3	1.07	(0.93-1.24)	1.12	(1.00-1.26)
4-3	1.24	(1.00-1.54)	1.36	(1.14-1.61)
1-4	1.18	(0.57-2.21)	1.70	(0.92-2.95)

2-4	1.42	(0.94-2.09)	1.29	(0.91-1.78)
3-4	1.03	(0.82-1.27)	1.19	(1.00-1.41)
4-4	1.20	(1.04-1.37)	1.42	(1.28-1.58)

Adjusted for age, wave, region, Partner in household, education, household income, and history of diseases.
OR = Odds ratio, CI = confidence interval.

Supplementary Figures

Supplementary Figure 1 – Directed Acyclic Graph (DAG) incorporating relevant and available covariates that were chosen for adjustment in the regression analyses.

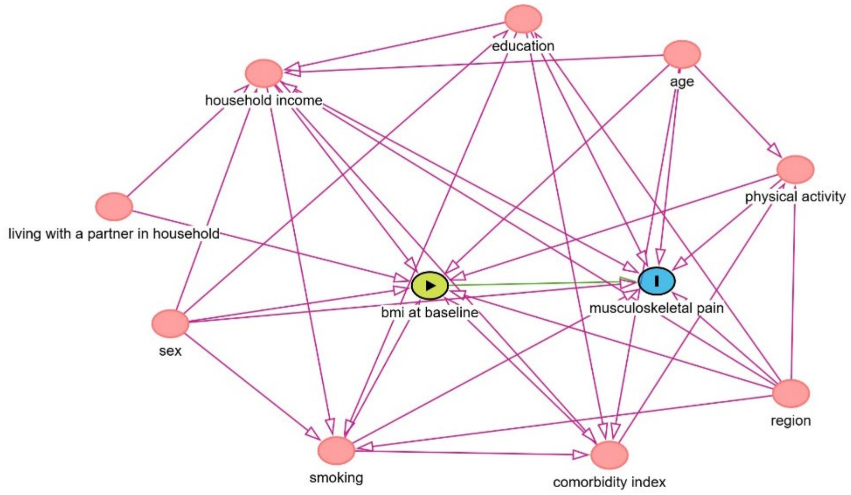

Supplementary Figure 2 - Longitudinal associations between BMI and the odds of back pain among middle-aged and older Europeans, based on their two most recent waves of participation in SHARE, stratified by sex.

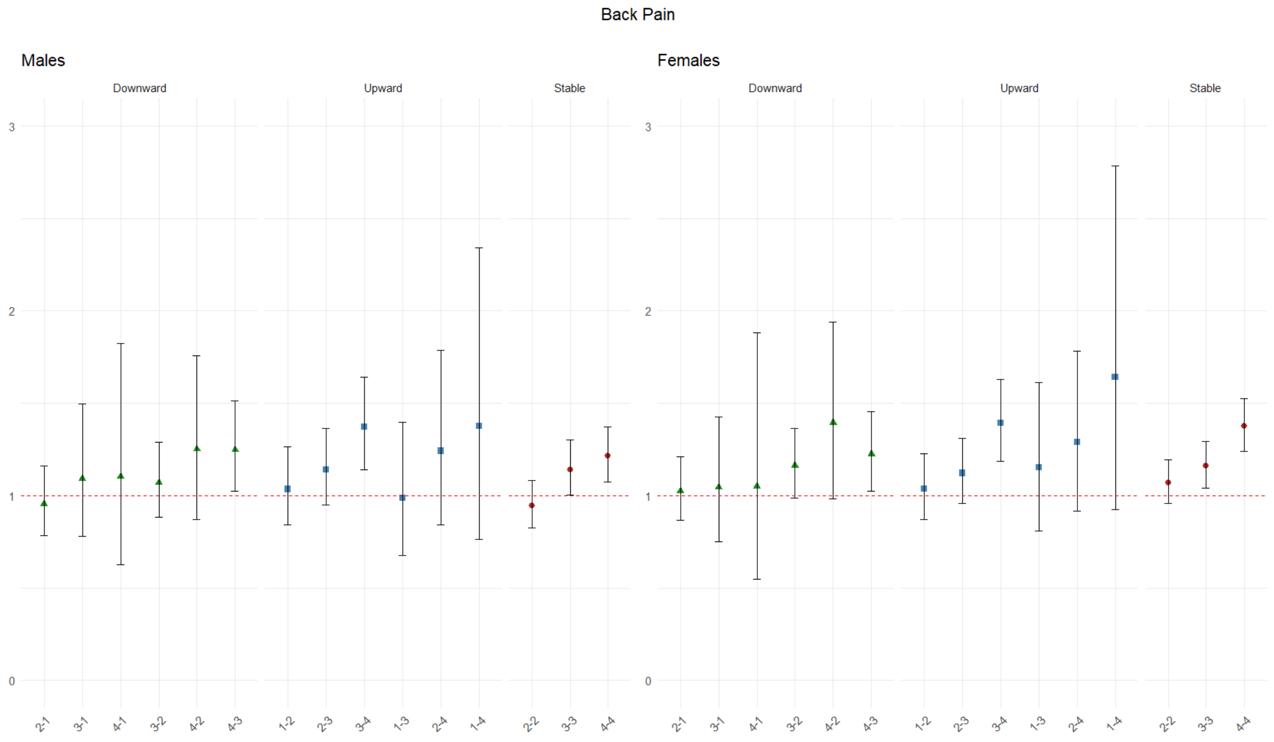

Supplementary Figure 3 - Longitudinal associations between BMI and the odds of back pain among middle-aged and older Europeans, based on their two most recent waves of participation in SHARE, stratified by sex.

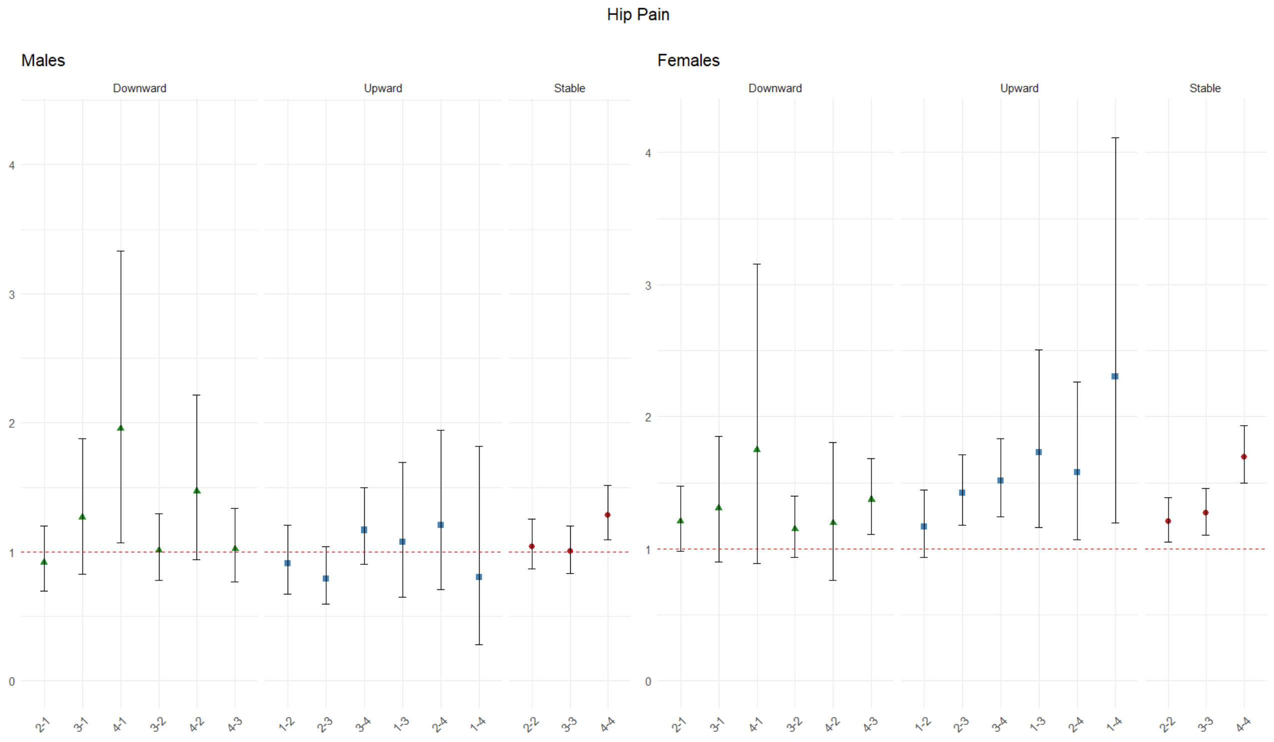

Supplementary Figure 4 - Longitudinal associations between BMI and the odds of knee pain among middle-aged and older Europeans, based on their two most recent waves of participation in SHARE, stratified by sex.

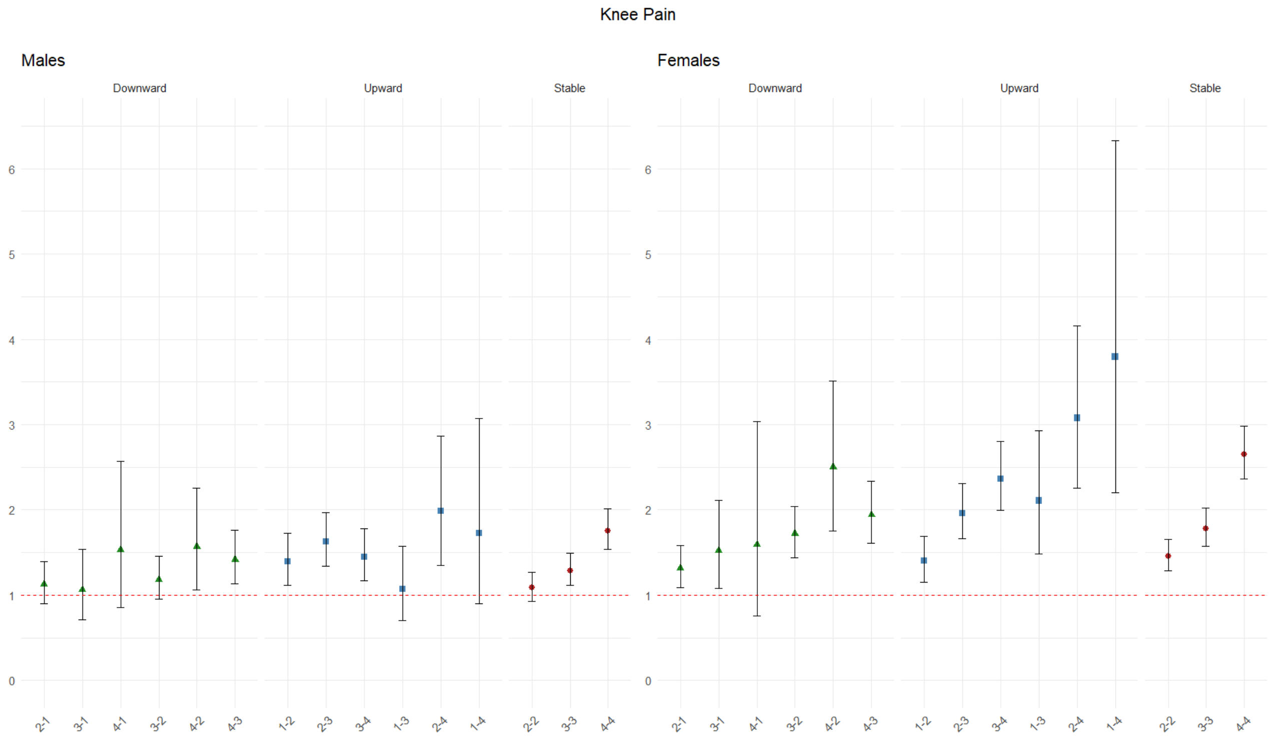

Supplementary Figure 5 - Longitudinal associations between BMI and the odds of other joint pain among middle-aged and older Europeans, based on their two most recent waves of participation in SHARE, stratified by sex.

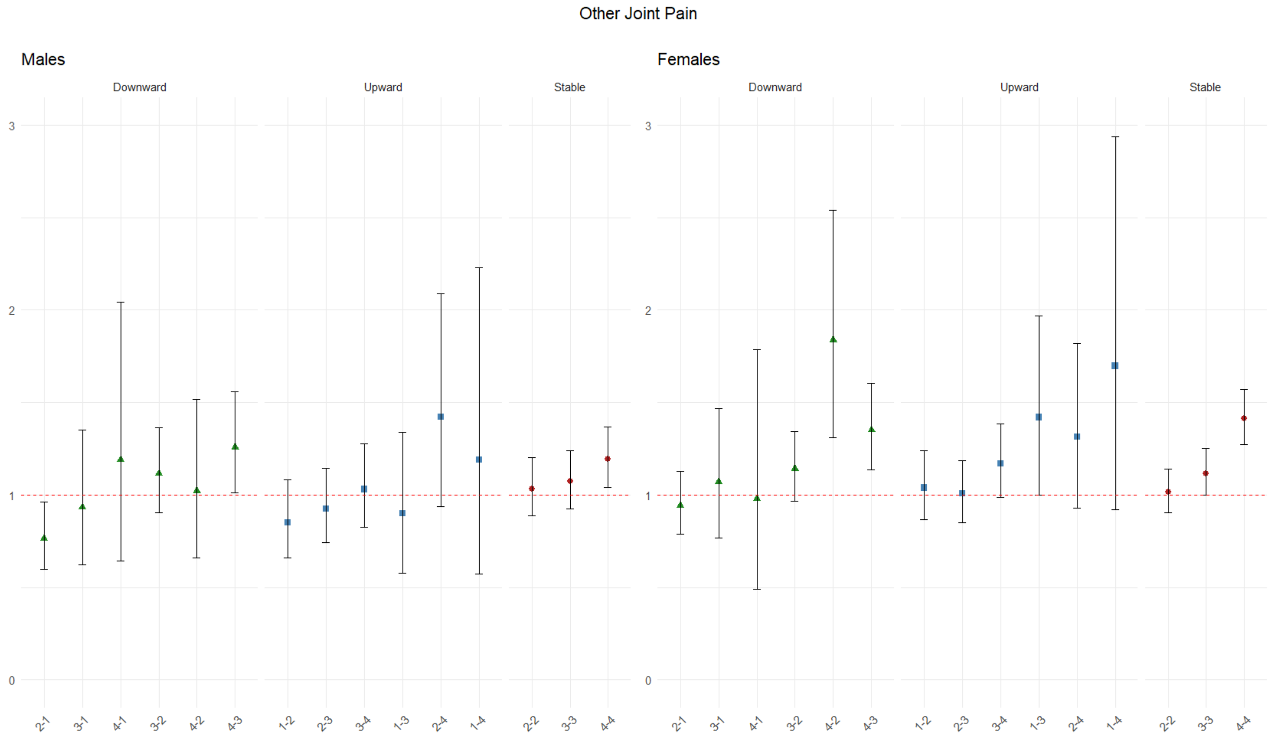

Supplement: Supplementary file 1 — Supplementary Material [file 41366_2026_2063_MOESM1_ESM.pdf]
